# Effects of radiation damage and inelastic scattering on single-particle imaging of hydrated proteins with an X-ray Free-Electron Laser

**DOI:** 10.1038/s41598-021-97142-5

**Published:** 2021-09-09

**Authors:** Juncheng E, Michal Stransky, Zoltan Jurek, Carsten Fortmann-Grote, Libor Juha, Robin Santra, Beata Ziaja, Adrian P. Mancuso

**Affiliations:** 1 European XFEL, Holzkoppel 4, 22869 Schenefeld, Germany; 2 Institute of Nuclear Physics, Polish Academy of Sciences, Radzikowskiego 152, 31-342 Krakow, Poland; 3 Center for Free-Electron Laser Science CFEL, Deutsches Elektronen-Synchrotron DESY, Notkestr. 85, 22607 Hamburg, Germany; 4 The Hamburg Centre for Ultrafast Imaging, Luruper Chaussee 149, 22761 Hamburg, Germany; 5 Max Planck Institute for Evolutionary Biology, August-Thienemann-Straße 2, 24306 Plön, Germany; 6 Institute of Physics, Czech Academy of Sciences, Na Slovance 2, 182 21 Prague 8, Czech Republic; 7 Institute of Plasma Physics, Czech Academy of Sciences, Za Slovankou 3, 182 00 Prague 8, Czech Republic; 8 Department of Physics, Universität Hamburg, Notkestr. 9-11, 22607 Hamburg, Germany; 9 Department of Chemistry and Physics, La Trobe Institute for Molecular Science, La Trobe University, Melbourne, VIC 3086 Australia

**Keywords:** Atomic and molecular interactions with photons, Single-molecule biophysics, X-rays

## Abstract

We present a computational case study of X-ray single-particle imaging of hydrated proteins on an example of 2-Nitrogenase–Iron protein covered with water layers of various thickness, using a start-to-end simulation platform and experimental parameters of the SPB/SFX instrument at the European X-ray Free-Electron Laser facility. The simulations identify an optimal thickness of the water layer at which the effective resolution for imaging the hydrated sample becomes significantly higher than for the non-hydrated sample. This effect is lost when the water layer becomes too thick. Even though the detailed results presented pertain to the specific sample studied, the trends which we identify should also hold in a general case. We expect these findings will guide future single-particle imaging experiments using hydrated proteins.

## Introduction

X-ray free-electron lasers (XFELs) provide X-ray pulses both of ultrahigh peak brightness and, simultaneously, of ultra-short pulse duration ranging from a few up to a few tens of femtoseconds. Single-particle imaging (SPI) experiments, performed at various XFEL facilities, aim to exploit these ultrabright, ultrashort pulses to determine the structure of single, non-crystalline biological molecules^1^. These experiments continue to improve their resolution^2–4^. However, a key goal, i.e., the resolution at length scales of a few Angstroms ($10^{-10}$ m), has not yet been realized.

One fundamental obstacle is the X-ray induced radiation damage. i.e., the rapid charging of the sample, induced by photoionization and subsequent secondary processes. The radiation chemistry of protein-water systems has been studied extensively^5,6^. However, results of these earlier experiments and calculations characterize a radiolytic behavior of the systems on nanosecond (and longer) timescales. In contrast, XFEL pulses give access to damage processes on femtosecond timescales. Radiation damage reduces a sample’s scattering strength on the few femtosecond timescale and triggers its subsequent disintegration on the few tens of femtoseconds timescale. Following the analysis performed in^1^, only intense and ultrashort X-ray pulses can image the sample before radiation-induced damage will significantly alter and ultimately destroy it. However, radiation damage is not the only limiting factor for SPI. Weak scattering from only a single molecule also contributes to the challenge of interpreting SPI data. Therefore, during an SPI experiment, a large number of two-dimensional diffraction patterns (many thousands, or perhaps even millions) from ‘identical’ particles (e.g. molecules, clusters or viruses) need to be recorded, in order to provide sufficient statistics to reconstruct a ‘meaningful’ average particle. Since the orientation of the sample with respect to the beam and the detector is unknown, the individual patterns must be oriented and merged into a three-dimensional diffraction volume (using dedicated algorithms)^7,8^ before the three-dimensional electron-density map is reconstructed via phase retrieval^9^. These limitations of X-ray FEL imaging to resolving the structure of biologically relevant single macromolecules in a SPI experiment have been discussed in detail by, e.g., Fortmann-Grote et al.^10^. In addition to those challenges, the heterogeneity of the protein sample may also limit high resolution single-particle imaging^11,12^. Previous studies^13^ have shown that a water tamper layer inhibits protein unfolding, which may occur in vacuum environments common in X-ray instruments. Further more, such a layer may also serve to keep the protein sample in a more restricted range of conformations^12^, which in turn could be expected to ease analysis.

As numerous diffraction patterns are needed for a successful 3D reconstruction of a macromolecule^14^, here the advantage of the FEL facilities based on the superconducting technologies—and hence offering high repetition rate X-ray pulses—can be seen. Among such facilities are: the operating European XFEL^15^ as well as the currently under construction LCLS II^16^, and the Shanghai High Repetition Rate XFEL^17^. The superconducting accelerator technology enables generating from tens of thousands up to a million light flashes per second, which, in turn, makes it possible to record the required high number of diffraction images within a feasible experiment duration.

**Figure 1:**
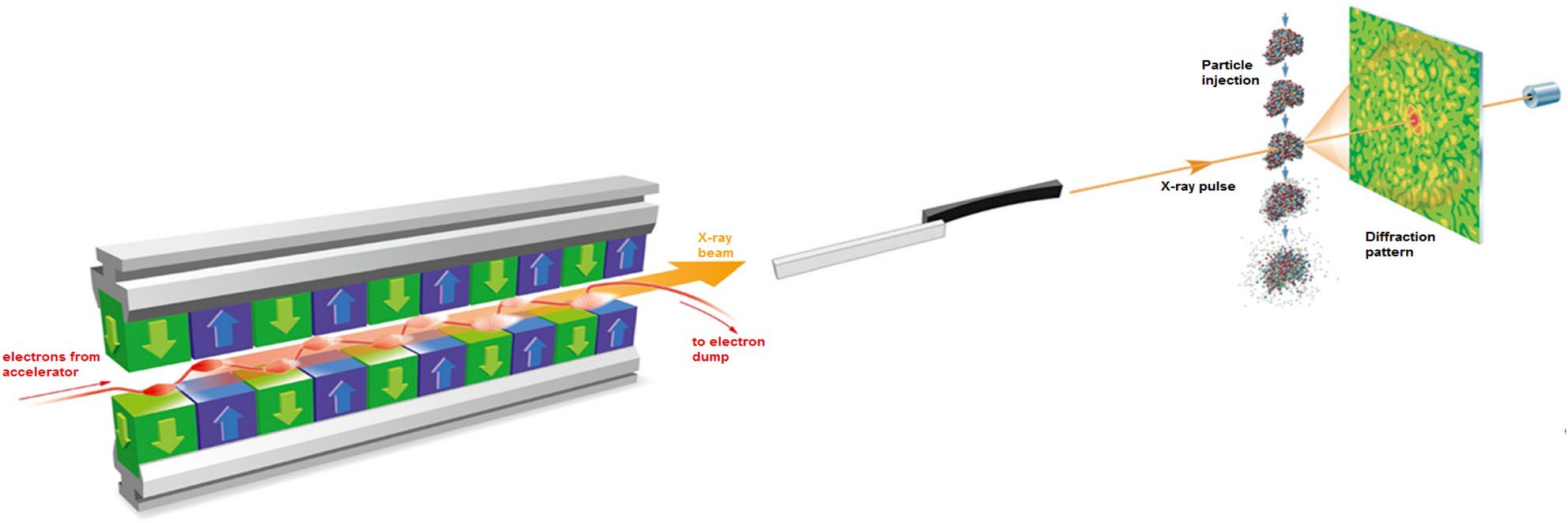
The schematic of a typical single-particle imaging experiment, modeled within our start-to-end simulation framework. X-rays propagate from the source to the sample through the beamline optics and then interact with the sample. The fraction of the beam scattered after the interaction is ‘captured’ by the detector in the end.

In order to explore the potential of the European XFEL for single-particle imaging, a comprehensive simulation platform SIMEX^18^ for SPI experiments has been developed^10,19^. This framework enables a realistic simulation of a single-particle imaging experiment at an XFEL facility, including source parameters, propagation of the coherent X-rays through optical elements, interaction of the photons with the imaged sample, detection of scattered photons, and structure determination (Fig. 1). The tool has a modular structure consisting of: (i) multidimensional simulation of the X-ray source; (ii) simulation of the wave-optics propagation of the coherent XFEL beams; (iii) atomistic modelling of photon-matter interaction; (iv) simulation of the time-dependent diffraction process, including incoherent scattering; (v) assembling noisy and incomplete diffraction intensities into a three-dimensional data set; and (vi) phase retrieval to obtain structural information^19^. The platform has been used in reference^10^ to estimate the optimal pulse duration for an X-ray pulse of 5 keV photon energy, feasible at the SPB/SFX instrument of the European XFEL, to image reproducible, biological molecules. Those simulations indicated an optimal pulse duration of around 9 fs.

Here we use SIMEX to investigate the potential advantage of using hydrated samples for single-particle imaging. That is, we consider a sample surrounded by a layer of water at the time of exposure to an ultrabright X-ray beam. This idea has been proposed by Hau-Riege et al.^20^ for single-particle diffraction imaging and further explored in earlier works^12,21–23^. Ideally , if an object of interest is covered by a (sufficiently thick) tamper layer of another material, this may slow the expansion of the object during its imaging. Specifically, during irradiation this outer (tamper) layer will be strongly ionized and expand quickly due to Coulomb repulsion between ions.

Plasma electrons will be attracted towards the central part of the sample, forming with ions a net neutral core. Therefore, an object located within the net neutral core (ideally the sample under study) will expand more slowly, as the expansion of the neutral core will be hydrodynamical only^20^.

However, this picture misses the effect of possible additional electronic damage of the hydrated sample due to its impact ionization by electrons originating from the tamper layer^23^. These electrons can contribute to the fast charging of the sample under study which would in turn result in lowering the diffractive scattering strength of the sample. As analyzed by Ziaja et al.^23^, the contribution of the electronic damage depends on the composition and size of the tamper layer.

Therefore, when considering the use of hydrated samples for single-particle imaging, one has to consider the overall effect of the water tamper. Our aim is to study the quantitative effect of physical processes that degrade the achievable “quality” (here resolution) of images during ultrashort pulsed X-ray imaging of hydrated proteins at realistic conditions, i.e., close to achievable experimental conditions today. Excluded from this study is the effect of practical, experimental limitations to image quality such as signal-to-background at detector, number of frames required for successful analysis and other such experimental parameters that are the subject of a subsequent work. This work extends the focus of previous works which investigated specific aspects of imaging (e.g. X-ray triggered sample dynamics^12,20,21,23^, effect of water layer on diffraction patters of undamaged proteins^22^, effect of structural heterogeneity on diffraction patterns^12^). In this paper, we study single-particle imaging of a hydrated protein for the parameters of the European XFEL, performing a detailed analysis with the start-to-end simulation platform. In the next section we will discuss the physical processes that occur upon X-ray irradiation, including both those contributing to radiation-induced damage as well as those affecting the diffraction signal—in the context of the tamper technique. Later, we will present the results from the simulations of a small, example protein covered with water layers of different thickness. We provide an insight into the ionization dynamics and demonstrate how the presence of the water layer influences the resolution-dependent fidelity of reconstructing the protein alone, and then discuss its consequences for SPI. Finally, we present our conclusions.

**Figure 2:**
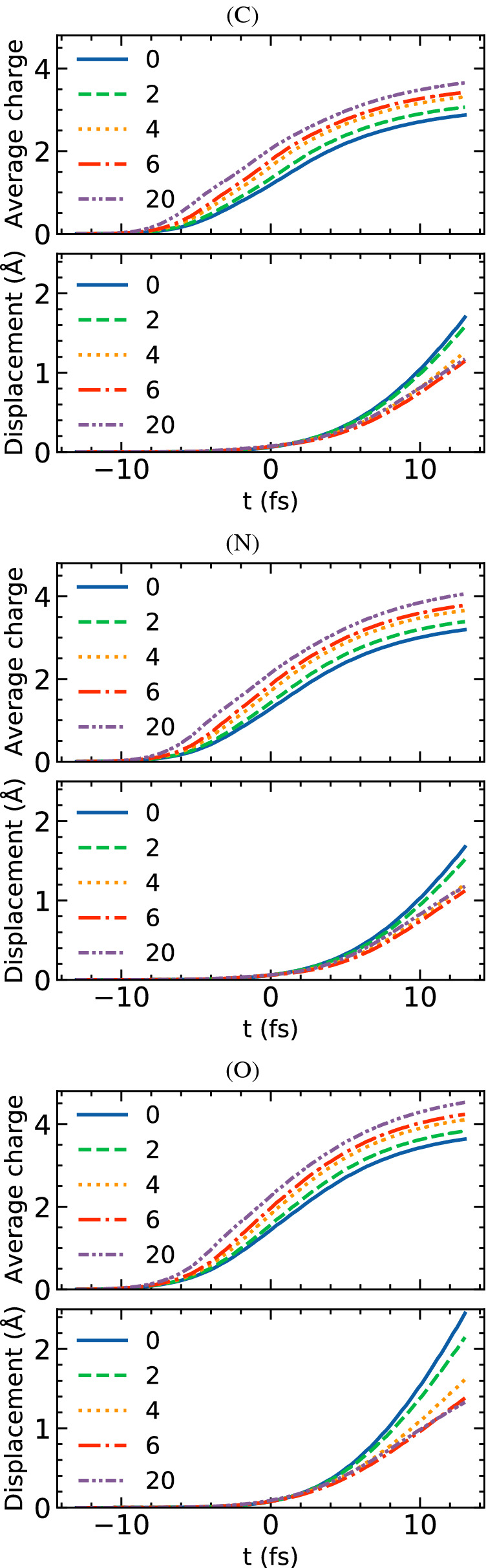
Transient average charge and transient average atomic displacement of carbon, nitrogen and oxygen within the 2NIP protein: with no water layer around and with 2–20 Å  thick water layer. Time zero corresponds to the maximum of the XFEL pulse intensity. The average quantities were obtained for 2712 carbon atoms, 735 nitrogen atoms and 859 oxygen atoms contained in the protein after 100 runs of XMDYN.

## Mechanisms of X-ray induced radiation damage

Irradiation with X-rays delivers energy to an illuminated material through photoabsorption. Photo- and Auger electrons released from deeply-lying atomic shells and the valence band ionize the material further through impact ionization. As a consequence, further electrons are released in collisional processes which trigger electron cascades. The fastest electrons are able to leave the sample, increasing the net charge of the sample. This increased positive charge attracts electrons towards the central part of the sample. A layer structure consisting of a core of approximately zero net charge and a positively charged outer shell is formed^23–26^.

The outer shell (consisting of unscreened ions) expands rapidly, due to the Coulomb repulsion of ions. Within the core, the ion charges are screened by quasifree electrons, and the core expands slowly (hydrodynamically). Therefore, if, during imaging, the imaged object was covered by a layer of another material of appropriate thickness, this layer would expand rapidly, whereas the object located within the net neutral core would expand much more slowly.

While atomic displacement becomes slower in the neutral core, advantageous to structure determination, the additional impact ionization by electrons from the tamper layer may enhance the ionization of the core when compared to the case without a tamper layer. This increased electronic damage would cause, e.g, earlier bond breaking. Also, as the signal carrying structural information in single-particle imaging is produced by elastic scattering of X-ray photons from bound electrons of atoms and ions, the presence of highly charged ions within a sample would lower this signal. The latter is simply due to the highly stripped ions having lower scattering factors. Also, the X-ray scattering from many additional plasma electrons present within the imaged sample would contribute to the signal as a background, reducing contrast in the scattering pattern.

Here, we will consider a specific tamper: a water layer. Water naturally tends to surround biological molecules, and is compatible with the delivery of biosamples to an X-ray beam. It consists only of light elements; therefore, one can expect that the effect of the electronic damage will not be so strong as in the case of a layer including heavy elements. In the latter case, the ionizing radiation would excite many electrons in the layer, which, in turn, would strongly increase the electronic damage in the imaged sample.

In the next section we will analyze the effect of water layer on the imaging quality of a hydrated, example protein.

**Figure 3:**
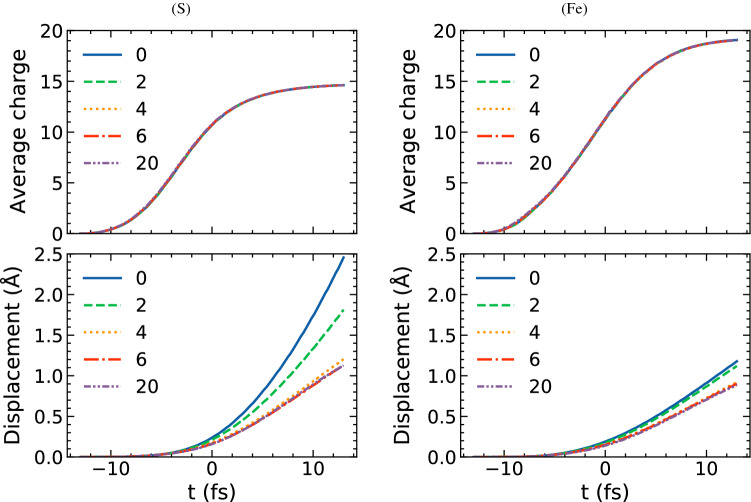
Transient average charge and transient average atomic displacement in sulphur and iron atoms within the 2NIP protein: with no water layer around and with 2–20 Å  thick water layer. Time zero corresponds to the maximum of the XFEL pulse intensity. The average quantities were obtained for 46 sulphur atoms and 4 iron atoms contained in the protein after 100 runs of XMDYN.

**Figure 4:**
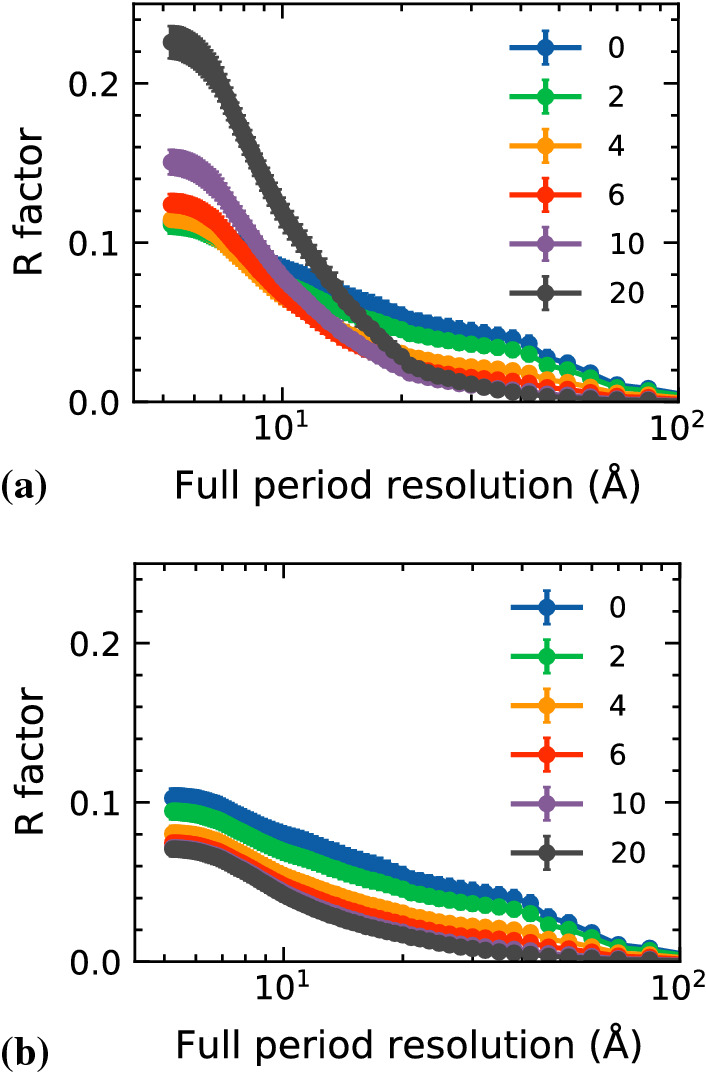
Measure of the diffraction pattern quality, R factor as a function of resolution. It was calculated, including: (**a**) elastic scattering from protein and inelastic scattering from water and protein; (**b**) only elastic scattering from protein. The error bars are the standard deviation from averaging over different orientations.

**Figure 5:**
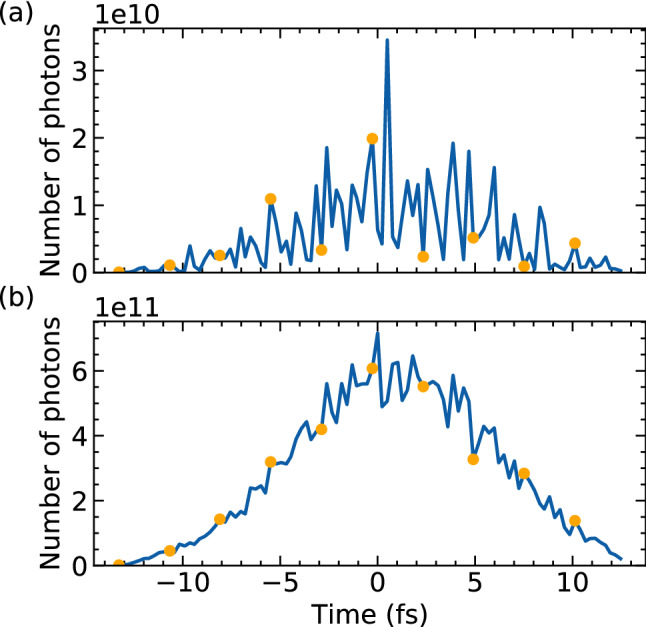
(**a**) A typical SASE beam temporal profile used in our simulation. (**b**) The temporal profile summed over the 55 SASE profiles. Time zero is at the maximum of the integrated profile in (**b**). The orange dots indicate the time slices used for the time integration of diffraction pattern.

## Start-to-end simulations of hydrated 2NIP protein

### Simulation scheme

We studied single-particle imaging of hydrated proteins using the example of 2–Nitrogenase–Iron Protein (2NIP) in a virtual, typical SPI setup (Fig. 1). 2NIP is a macromolecule containing $\sim 8700$ atoms (including hydrogen atoms), of an approximate diameter of 70 Å along the longest axis. The same protein has been used for our earlier studies^10,19^. We explore a range of plausible water layer thicknesses in the calculations performed—0, 2, 4, 6, 10 and 20 Å (for details see ‘Methods’).

The X-ray pulse parameters were chosen based on the parameter optimization study performed in^10^ for the SPB/SFX instrument of the European XFEL. The photon energy was 5 keV, and the pulse length was 9 fs FWHM. The SASE (Self-Amplified Spontaneous Emission) source wavefronts were taken from the XFEL Photon Pulses Database (FAST-XPD: https://in.xfel.eu/fastxpd/) and then propagated using the Fourier optical wave propagation code WPG^27,28^ through a modeled version of the SPB/SFX instrument^19^.

The photon-matter simulations have been performed with the molecular-dynamics code XMDYN^29–32^. This mixed quantum-classical simulation tool provides detailed information on the actual ion and electron content in the irradiated sample, including actual atomic configurations, positions of atoms and released free electrons, and particle energies as a function of simulation time. For this work, we have applied no photon or detector noise in the generated patterns as we focus here on fundamental limitations. The practical aspects, including sensitivity to noise, number of patterns required, etc. will be explored in a future, more technical work. The diffraction patterns were calculated with SingFEL^33^ via a module integrated in the SIMEX platform^18^. For more details on the calculations, see ‘Methods’.

### Results and discussion

For each of 6 water layer thicknesses, 1000 different XMDYN trajectories were calculated, assuming various spatial orientations of the hydrated protein in the beam. For each trajectory, 20 time-integrated two-dimensional diffraction patterns were generated. By comparing the output diffraction (including the effects of radiation damage in the presence of a tamper layer) to that of a readily-calculated, ideal, radiation-damage-free diffraction pattern, we evaluate the effect of each considered tamper layer on the quality of the diffraction data measured. The key question addressed by the results below is to identify an optimal thickness of the water layer at which the attainable resolution for imaging the hydrated sample becomes the highest.

Below we show the theoretical predictions of X-ray induced dynamics in the sample, in particular, on the transient average ion charge and transient average atomic displacement for the 5 constituent elements of the protein 2NIP relevant in X-ray imaging, i.e., carbon, oxygen, sulphur, iron and nitrogen (Figs. 2 and 3). Time zero in the figures is defined at the maximum of the integrated temporal X-ray profile in Fig. 5(b). For oxygen, we consider only the atoms contained within the protein, not those contained within the water layer. Also note that although the displacement of H atoms is large (not shown), these atoms are effectively ‘invisible’ for the X-ray imaging studied here. This is due to their low electron density which implies that their contribution to the diffraction data is very small. It is a similar approximation as used in the previous work^20^. Therefore, we do not describe them in this paper.

Calculations of the ionization dynamics obtained for the protein covered with water layers of different thicknesses (2–20 Å) are compared to those obtained with no water layer. This enables us to analyze the reduction of the atomic displacement and the effect of the electronic damage in the hydrated sample.

Carbon, nitrogen and oxygen atoms are spread approximately uniformly throughout the protein sample. If the protein is covered with a water layer, the protein atoms adjacent to the surface are subject to an increased ionization, when compared to the case of the protein without a tamper (Fig. 2). This is due to the electrons released from the surrounding water layer. They penetrate into the protein sample, and enhance ionization of C, N and O atoms therein via collisional processes.

However, this is not the case for a relatively few S and Fe atoms (46 and 4, respectively) in Fig. 3. Their transient average charges, both with and without a water layer, are almost the same. Our calculations show that these heavy atoms are predominantly ionized in photoionization events and consecutive inner-shell Auger decays. For these species, the impact ionization events constitute only a few percent (up to 5%) of the total number of ionizing events. This is due to the fact that we consider here a finite-size sample. Therefore, energetic electrons released in photoinduced processes can leave the hydrated protein, inducing only a smaller number of collisional ionization events. This is in a strong contrast to the case of protein nanocrystals^32^, where the majority of the photo- and Auger electrons remain in the sample, ionizing heavy atoms heavily.

As we discussed above, charging of the X-ray irradiated sample ultimately has an impact on the single-particle imaging quality through two major effects: (i) the progressing atomic displacement and (ii) the decrease of scattering factors of individual atoms due to their ionization. They both affect the elastic scattering component of the diffraction signal. However, the increasing sample ionization also changes the inelastic scattering component, strengthening the signal from plasma electrons. To quantify the radiation-damage induced change of imaging quality, we use the R factor metric, known from crystallography^34^. It has been previously used to compare non-ideal to ideal single-particle diffraction data^1,20,35,36^. The factor R takes a value between 0 and 1. For identical patterns, $R=0$. A value of $R=0.2$ seems to be a well-accepted empirical cutoff for ‘similar enough’ in both crystallography and SPI. In this paper, the reference dataset is represented by the undamaged protein with all water molecules removed. For details on the R factor calculation, see ‘Methods’ and the references^37^.

For a measured, experimental scenario, the best-case expectation is that one can separate the elastic signal of the protein from the elastic signal of the water layer during reconstruction of the combined water-particle system. This cannot, in general, be done for the inelastic signal, which includes the contributions from both bound and free electrons originating from the whole sample. As such, for this study we consider the diffraction data without the (large) contribution of the elastic scattering from the water layer. For a further discussion of the validity of this approach, see the Supplementary Material.

The behavior of the R factor in Fig. 4(a) varies across both the higher resolution region ($D \le 10$ Å) and low resolution region ($D \ge 20$ Å). Figure  4(a) shows clearly that there is a trade-off in imaging ‘quality’ as measured by the R factor for the two different regimes. Specifically, thicker tamper layers result in lower R values for larger feature sizes (low resolution case), while thinner layers perform better for smaller feature sizes (high resolution case). The optimal range of water tamper thicknesses, which perform better in terms of resolution than the case of protein without water layer lies between 2 Å and 6 Å.

In the high-resolution region, the R factor in Fig. 4(a) is dominated by the effect of inelastic scattering (from the whole sample). The inelastic scattering component to the signal grows with increasing water layer thickness, as the total number of scatterers, both bound and free ones, then increases. Consequently, the R factor becomes the largest for the sample with 20 Å thick water layer in the high resolution region. Note that for the elastic scattering component of the signal the trend is opposite, as Fig. 4(b) shows: the R factor decreases as the water layer thickness increases. The main reason is the reduction of ion charge fluctuations when thicker layers of water tamper are present. For further details, see the Supplementary Material.

In the low-resolution region, the R factor values in Fig. 4(a) (including both elastic and inelastic signal) and Fig. 4(b) (including elastic signal only) show almost no difference for each water layer thickness. This confirms that—as expected—the inelastic scattering has almost no influence on the total diffraction signal in the low-resolution region. As we show in the Supplementary Material, in this region the behavior of R factor is dominated by the spatial inhomogeneity of ionization within the sample which becomes less pronounced with increasing thickness of the water tamper).

In the transition region between the resolutions of $\sim$10 Å and $\sim$20 Å, the high and low resolution trends interchange in case of both elastic and inelastic signal recorded (Fig. 4(a)). For the elastic scattering case (Fig. 4(b)), there is a continuous decrease of the R factors for all different water thicknesses.

We emphasize that in this current, best-case study of the 2NIP protein (i.e., assuming successful subtraction of elastic signal from the water layer, no detector noise etc.), the R factor remains below the value of 0.2 for almost all water layer cases (except for the 20 Å water layer case at a few Å resolution), confirming that structural information is preserved within diffraction patterns with a good fidelity. However, the fundamental trends we identify here—that there is a trade-off in optimal tamper layer thickness depending on which resolution range is prioritized—are expected to also hold in less ideal cases, where R factors may be overall higher.

## Conclusions

We have explored the effect of water layer thickness on the fidelity of diffraction patterns produced from hydrated proteins in a modeled single-particle imaging experiment. This model has included parameters relevant to a real XFEL experiment as well as an element-sensitive, time-resolved treatment of radiation damage performed with a dedicated molecular dynamics simulation tool. All cases studied here have relied on the subsequent removal of the elastic scattering component originating from the water layer from the total signal as well as neglecting the experimental noise—meaning that the results presented here represent the best-case scenario. The details and practicalities of such a procedure will be the subject of future work.

For the protein studied here, 2NIP, we have seen that thicker tamper layers (10 Å and 20 Å) serve to limit the effects of radiation damage at the low resolution region at the expense of the highest resolution region. Thinner water layers (of a few Å thickness) behave the opposite, and preferentially ‘protect’ the highest resolution (smallest) features. A similar qualitative behavior is expected to occur in general for other samples of similar size.

Such a result is encouraging for experimental SPI, where there may always be a few-Å thick water layer present on any practical sample, due to the aqueous environment used for sample delivery. Although it is difficult to measure the actual tamper layer thickness in experiments, there have already been some efforts to reduce the sample droplet size with the electrospray technique^38^, which may permit access to an optimal range of water layer thickness in future. Therefore, our observations are expected to have implications for future single-particle imaging experiments using hydrated proteins.

## Methods

### Water layer generation

The input configurations for our simulations were taken from our previous work^37^. Therein, the water layer was relaxed to reach thermal equilibrium, using an MD simulation with the Langevin thermostat at $T = 293$ K. After reaching the equilibrium, 125 snapshots separated about 640 fs apart were recorded. That yielded water molecule ensembles with negligible spatial correlation between the snapshots.

The water molecules were generated within a simulation box of dimensions $27.3 \times 26.1 \times 27.9$ nm$^{3}$ so that the 2NIP protein^39^ obtained from rcsb.org was initially surrounded by 10 nm of water in each axis direction. The MD simulation package NAMD^40^ was utilized for the MD equilibration of the water layer with periodic boundary conditions. Energy minimization was then applied, using the TIP3P force field^41^ for the water-water interactions and the CHRMM22 force field^42,43^ for the water-protein interactions, respectively. The constituent atoms in the protein were fixed during the energy minimization process, only the water molecules in the layer were allowed to move, until the energy converged^44^.

To reach equilibrium, the aforementioned water-protein system was kept at the temperature of 293 K using a Langevin thermostats for 50,000 MD steps of 2 fs stepsize. The details were reported in a previous conference^37^.

In the end, the water layer was trimmed to various thicknesses of 2, 4, 6, 10 and 20 Å.

### XFEL source and wave propagation

The XFEL Photon Pulses Database, driven by the *FAST* code^45^ and operated by the European XFEL, provides precomputed pulses at the undulator exit for a large range of accelerator energies, bunch charges, undulator lengths and photon energies at the European XFEL. For the current simulation, 55 different simulated SASE pulse profiles were generated with 4.96 keV photon energy and a full duration at half maximum (FDHM) of 9 fs from 12 GeV electrons with electron bunch charges of 100 pC^19^. The SASE pulses are then propagated using the Fourier optical wave propagation code *WPG*^28^ powered by *SRW*^27^ in the same SPB/SFX instrument setup described by C. H. Yoon et al.^19^. Each pulse has approximately $5 \times 10^{11}$ photons after propagation. The nominal focus size is $250 \times 160$ nm$^2$ FWHM, yielding the fluence of $9.7 \times 10^5$ J/cm$^2$ and the intensity of $1.1 \times 10^{20}$ W/cm$^2$. Fig. 5(a) shows a typical temporal profile of a single SASE pulse used for our diffraction simulation.

Let us emphasize that realistic pulses from the wave propagation simulation represent real experimental conditions more closely than an ideal Gaussian pulse. The main benefit is that variations in the fine spiky temporal profile of an XFEL pulse can introduce some change in the ionization dynamics compared to a smooth Gaussian temporal profile, even in the case of isolated atoms (see, e.g., the paper by Rohringer et al.^46^), especially for short pulses (< 10 fs). This will affect the diffraction pattern, even if it is time integrated. Using as realistic temporal profiles as feasible allows this (physical) effect to manifest for the subsequent evaluation of diffraction data.

### Molecular dynamics simulations with XMDYN code

For the radiation simulation, we used XMDYN, a molecular-dynamics (MD) and Monte-Carlo-based code for modeling X-ray driven dynamics in complex systems. Coupling on-the-fly to the atomic structure calculation tool XATOM^29,36,47^ enables XMDYN to provide a microscopic description of the X-ray induced processes in matter, such as atomic photoionization, inner-shell Auger and fluorescent decay, collisional ionization, recombination, and the real space dynamics of the atoms and quasi-free electrons emitted. XMDYN simulations follow the temporal evolution of a stochastically ionized system. The code has been successfully applied to simulate the interaction of X-rays with clusters and macromolecules^29–32^.

Following the stochastic ionization dynamics of the sample (with the propagated beam described in the previous section, the real space positions of the atoms, the atomic form factors for elastic scattering, and the structure factors for inelastic scattering were calculated^48^. For each specific water layer thickness as well as for the case without water layer, 1000 MD runs were performed, within which the 55 propagated beam profiles were taken alternately. For each run, a random rotation of the sample was performed, and the rotation quaternion recorded. 100 snapshots covering the whole 26 fs simulation time span were recorded for further analysis.

As the XFEL pulses are linearly polarized, and the orientation of the protein under the experimental conditions is random with respect to the X-ray polarization vector, in a separate, dedicated simulation we tested the effect of the initial X-ray polarization on the ion charge and the ion displacement distributions (not shown). As the angular distribution of photoionized electrons depends on the polarization direction, this could eventually influence the distribution of charges and the ion displacement. However, the results show that the dependence of those observables on the initial orientation of the molecule in respect to the polarization vector is negligible. This allowed us to reduce the number of XMDYN simulations needed to perform the analysis presented in this paper, lowering the computational costs of this anyway computationally expensive study.

### Diffraction simulations

Using the X-ray temporal intensity profiles from the wave propagation simulation and atomic positions with corresponding scattering factors from the previous sections, we calculated the 2D diffraction patterns with the ‘pysingfel’ module^10,19,33^ integrated in the SIMEX platform^18^. We took 20 time-integrated diffraction patterns for each XMDYN time-resolved trajectory, making a total of 20,000 diffraction patterns for each water layer thickness. We generated a rotation quaternion list, so that the overall diffraction orientations for each water layer thickness followed the same uniform distribution over the SO(3) rotation group, with each quaternion corresponding to one diffraction pattern orientation. For each diffraction pattern, we rotated the atomic positions of each XMDYN trajectory based on the quaternion recorded in the XMDYN simulation to reach the final orientation defined in the quaternion list, the same way for each water layer thickness. The diffraction patterns were then integrated with incident X-ray pulse weighted within the integral interval over the pulse duration. For a given electronic configuration, the number of time-integrated scattered photons included both the coherent (elastic) scattering signal from bound electrons and the incoherent (Compton) scattering signal from bound and free electrons as shown in Eq. (1)^10,19^:

1 $$\begin{aligned} n(\mathbf{q} ) = \Omega \frac{{\mathrm{d}}\sigma _T(\theta )}{{\mathrm{d}} \Omega }\sum _{i} n_{\text{in}}(t_i)[|F(\mathbf{q} ,t_i)|^2+S(\mathbf{q} ,t_i)+N_{\text{free}}(t_i)], \end{aligned}$$

where $\mathbf{q}$ is the scattering vector, $\Omega$ is the solid angle spanned by the respective detector pixel pointed by the corresponding vector $\mathbf{q}$, ${{\mathrm{d}}}\sigma _T(\theta )/{\mathrm{d}} {\Omega }$ is the differential Thomson cross section, $F(\mathbf{q},t)$ is the structure factor for elastic scattering, $S(\mathbf{q},t)$ is the static structure factor for inelastic (Compton) scattering, and $N_{\mathrm{free}}(t)$ is the number of free electrons^48^. The time $t_i$ corresponds to the *i*th XMDYN snapshot, and $n_{\mathrm{in}}(t) = \sum _{t\le t_j<t+\Delta t} \,n_0(t_j)$ is the number of incident photons in a user-defined time span $\Delta t$, where $n_0(t)$ is the number of XFEL photons as a function of time obtained in the wave propagation simulation.

In our study, we chose the $\Delta t$ to be $\Delta t = 10 \times \mathrm{timestep}$. It was 0.27 fs, as described in the molecular dynamics simulation section. The detector is represented by a $81 \times 81$ pixel array with the pixel size of 1200 μm. The sample to detector distance is 13 cm. The full-period resolution is approximately 7 Å at the detector edge.

### R factor analysis

Following the definition in^36^, the R factor assessing the deterioration of diffraction patterns is defined as:

2 $$\begin{aligned} R(D) = \sum\limits_{{q \le 2\pi /D}} {\Omega ({\mathbf{q}})\left| {\frac{{\sqrt {N({\mathbf{q}})} }}{{\sum\limits_{{q^{\prime } \le 2\pi /D}} {\sqrt {N({\mathbf{q}}^{\prime } )} } \Omega ({\mathbf{q}}^{\prime } )}} - \frac{{\sqrt {N_{{ideal}} ({\mathbf{q}})} }}{{\sum\limits_{{q^{\prime } \le 2\pi /D}} {\sqrt {N_{{ideal}} ({\mathbf{q}}^{\prime } )} } \Omega ({\mathbf{q}}^{\prime } )}}} \right|} , \end{aligned}$$

where $N(\mathbf{q}) = n(\mathbf{q}) / \Omega (\mathbf{q})$ is the number of photons per unit solid angle scattered from the sample undergoing radiation damage (with $n(\mathbf{q})$ being the number of scattered photons on a pixel whose scattering vector at the pixel center is $\mathbf{q}$), and $N_{ideal}(\mathbf{q})$ is the number of photons scattered from the reference (undamaged) sample. The parameter *D* is the considered resolution length scale determined by the detector geometry.

As described in the previous section, we calculate the diffraction patterns with the sample rotated to follow a uniform orientation distribution. For each orientation *k*, we calculate the R factor for the diffraction pattern $P_{j,k}$ from the damaged sample with various water layer thickness $j=0,2,4,6,10,20$ Å, compared with the pattern of undamaged sample without water layer $P_{\mathrm{ideal}, k}$, and then get an average R factor profile for each water thickness *j*. Notice that when we have the same sample orientation for two diffraction patterns to compare, the two patterns correspond to the same reciprocal space, i.e., the scattering vector of a certain pixel will be the same for the two patterns. Thus, to get the average R factor for each water layer thickness, we first calculate the R factors for the 2D diffraction patterns with the same orientation *k* and then get the average value of them as explained in Eq. (3):

3 $$\begin{aligned} \langle R(P_{j,k},P_{\mathrm{ideal}, k}) \rangle _k= \frac{1}{N_j}\sum _k R(P_{j,k},P_{\mathrm{ideal}, k}), \end{aligned}$$

where $N_j$ is the number of diffraction patterns with radiation damage for each water layer thickness. The results of this study are shown in Fig. 4.

## Supplementary Information

Supplementary Information.

## Data Availability

Data are available from the corresponding author upon reasonable request.
