# Supplementary material for: Effects of radiation damage and inelastic scattering on single-particle imaging of hydrated proteins with an X-ray Free-Electron Laser
Source: Sci Rep. 2021 Sep 9;11:17976. doi: 10.1038/s41598-021-97142-5 (PMC8429720; doi:10.1038/s41598-021-97142-5)
Supplement: Supplementary file 1 — Supplementary Information. [file 41598_2021_97142_MOESM1_ESM.pdf]

Supplementary material: Effects of radiation damage and inelastic scattering on single-particle imaging of hydrated proteins with an X-ray Free-Electron Laser

Juncheng E[1*], Michal Stransky[1,2*], Zoltan Jurek[3,4], Carsten Fortmann-Grote[1,5], Libor Juha[6,7], Robin Santra[3,4,8], Beata Ziaja[2,3*], and Adrian P. Mancuso[1*,9]

[1]European XFEL, Holzkoppel 4, 22869 Schenefeld, Germany
[2]Institute of Nuclear Physics, Polish Academy of Sciences, Radzikowskiego 152, 31-342, Krakow, Poland
[3]Center for Free-Electron Laser Science CFEL, Deutsches Elektronen-Synchrotron DESY, Notkestr. 85, 22607 Hamburg, Germany
[4]The Hamburg Centre for Ultrafast Imaging, Luruper Chaussee 149, 22761 Hamburg, Germany
[5]Max Planck Institute for Evolutionary Biology, August-Thienemann-Straße 2, 24306 Plön, Germany
[6]Institute of Physics, Czech Academy of Sciences, Na Slovance 2, 182 21 Prague 8, Czech Republic
[7]Institute of Plasma Physics, Czech Academy of Sciences, Za Slovankou 3, 182 00 Prague 8, Czech Republic
[8]Department of Physics, Universität Hamburg, Notkestr. 9-11, 22607 Hamburg, Germany
[9]Department of Chemistry and Physics, La Trobe Institute for Molecular Science, La Trobe University, Melbourne, VIC 3086, Australia
[*]Correspondence and requests for materials should be addressed to: juncheng.e@xfel.eu, michal.stransky@xfel.eu, ziaja@mail.desy.de, adrian.mancuso@xfel.eu

Explanation of R factor behavior in high resolution region

Let us first emphasize that Fig. 4(a) in the main text includes the inelastic signal both from water and protein. The justification is following. When analyzing the real experimental signal, we expect to be able to separate the elastic signal from the protein only from the elastic signal from water layer only, and subtract the latter as a background. This cannot be done for the inelastic signal, as it was explained in the section 'Results and discussion'. Therefore, in our current analysis we will include the total inelastic scattering signal from the water-and-protein sample.

The behaviour of R in Fig. 4(a) in the main text in high resolution region can then be explained by the effect of inelastic scattering. The inelastic scattering from the whole sample contributes stronger with the increasing water layer thickness, as the total number of scatterers, both bound and free ones, then also increases. Consequently, the R factor becomes the largest for the sample with 20 Å thick water layer at high resolution $D \leq 10$ Å.

This is confirmed by comparing the behavior of R in Fig. 4(a) with the behavior of R factor in Fig. 4(b) in the main text, the latter originating from elastic scattering on the protein only. Below we will show that in the latter case, the behavior of the R factor is mostly affected by ion charge fluctuations and to a smaller extent by atomic displacement. To prove this, in two following calculations we will separate the effects of the 'ionization damage' and the 'displacement damage' on the R factor.

In order to quantify the effect of the 'ionization damage' and to eliminate the effect of the 'displacement damage', in our first calculation we fixed atom positions to their initial positions. The resulting R factor is shown in Fig. S1. We can see that the R factor in Fig. S1 is very close to the R factor in Fig. 4(b) in the main text, where the overall damage has been included.

In order to evaluate the effect of the 'displacement damage' on the R factor, in our second calculation we fixed the atomic form factors to their initial values representing a neutral sample. Fig. S2 shows that the resulting R factor degradation occurs mostly in the high resolution regime, with a weak dependence on the tamper thickness. After comparison with Fig. 4(b), we realize that the 'displacement damage' has a smaller effect on the overall damage than the 'ionization damage'.

Fig. 2 in the main text shows the results on the average charge and the average displacement for C, N, O atoms in the protein, obtained as a function of time for various water layer thicknesses. Shortly after time-zero, the trend in the average displacement becomes visible. It becomes the largest for the sample without a water layer, and the smallest for the sample with the thickest water layer. As discussed in the reference[1], the presence of the outer water layer induces sample expansion near the sample center to become hydrodynamic and not Coulombic. However, for the chosen pulse duration the displacements are still relatively small (sub-angstrom) for most of the pulse duration. As demonstrated in Fig. S2, these small differences in the displacements observed for water layers of various thickness affect the R factor only weakly and only in the high resolution region.

To sum up, the behavior of the R factor in the high-resolution region as a function of tamper water layer thickness is then affected by two mechanisms: (i) inelastic scattering, and (ii) ion charge fluctuations. The latter mechanism starts to manifest for thinner water layers (thickness $\leq$ 4–6 Å), where the contribution of elastic signal prevails over that of the inelastic signal.

Explanation of R factor behavior in low resolution region

The low resolution region represents larger features of the imaged object. Therefore, in contrast to the trend in the high resolution region, ion charge fluctuations and tiny differences in displacements observed for water layers of various thickness will not affect the R factor in the low resolution region. In particular, the weak effect of the 'displacement damage' in the low resolution region is demonstrated in Fig. S2.

The question is, what effect can be responsible for the behavior of the R factor in the low resolution region? Fig. S3 shows the radial profile of the average number of bound electrons on carbon atoms in the irradiated 2NIP protein at the time zero. One can see that the average number of bound electrons per atom, $\langle Q(r) \rangle$, is not uniformly distributed in the sample, but increases towards the sample edge. For thicker water tampers, this quantity becomes more uniformly spread within the sample. The average charge can be fitted with an approximate relation:

$$\langle Q(r) \rangle = a + b \cdot e^{\frac{r-R}{L}}, \tag{1}$$

where r[Å] is the radial coordinate, measured from the particle center, and $R = 38$ Å is the approximate particle radius. For the case with no water layer, the radial profile of the number of bound electrons can be fitted with $a = 4.25, b = 1.37, L = 17$Å. Similar behavior can be observed for nitrogen and oxygen atoms within the protein (Fig. S3), and fitted by a similar equation with different coefficients.

The radial non-uniformity in $\langle Q(r) \rangle$ can be explained with the spatially non-uniform effect of impact ionization by plasma electrons. In the cases with thin or no tamper layer, some fraction of the released electrons close to the edge can leave the particle, locally reducing the plasma electron density. Therefore, there is less impact ionization in the vicinity of the sample edge, when compared with the sample center, where there are many (impact ionizing) trapped electrons. For thicker tampers, the electrons from the tamper layer can enter the sample and increase the ionization near the edge. As a result, the (increased) ionization is more uniformly spread across the sample.

In order to evaluate the effect of the non-uniform distribution of bound electrons on the R factor, a test calculation was performed. A spherical sample containing 4306 carbon atoms, randomly distributed at the density similar to non-hydrogenic atomic density of the protein was created. Non-uniformity in the bound electron distribution was introduced with the average number of bound electrons on carbon atoms given by equation (1), with parameters $a = 4.25, b = 1.37, L = 17$ Å for sample representing damaged protein without a water layer, $a = 4.30, b = 0.727, L = 6.7$ Å for sample representing damaged protein with a 4 Å water layer, and $a = 4.22, b = 0.387, L = 4.8$ Å for sample representing damaged protein with a 6 Å water layer. As a reference sample for the R factor calculations, the same sample of carbon atoms at the same positions was used, but with a constant number of bound electrons on all atoms, corresponding to that one at $r = 0$. The resulting R factors are shown in Fig. S4. Their behavior matches qualitatively the R factor behaviour in the low resolution range 20-100 Å in Fig 4(b), supporting our explanation that it is determined by the non-uniformity of bound electron distribution in the sample.

Justification for neglecting the elastic scattering from a water layer

The analysis performed in this work has excluded the diffraction signal due to the elastic scattering from the water layer around the sample, in order to better observe the effects of radiation damage and inelastic scattering on the diffraction signal. Figure S5 shows that, in practice, the elastic scattering signal from the water layer dominates, and increasingly so for thicker layers – as one would expect from considering the increasing number of atoms (scatterers) in thicker water layer(s). One could also expect that this signal may be reasonably removed in two limiting cases. The first is the case of a relatively thin layer of water, which is then reproducibly ordered around the sample particle. In this case, the hypothesis that the entire water-sample particle could be reconstructed as a whole, and the reconstructed water layer removed afterwards seems quite plausible.

The other extreme case is that of a (likely thicker) layer of water, which is assumed to be of uniform thickness in each case though otherwise not ordered around the sample. A diffraction pattern from such a sample would contain a 'sample' component, which would be consistent for any given orientation, as well as a 'water' component, which – apart from the size component due to its thickness – would vary randomly even for identical orientations. In the case that the structure internal to the water layer is not resolved (i.e., the resolution is poorer than about the size of a water molecule), the composition of the three-dimensional diffracted intensity from the water-sample particle would be expected to yield 3D intensities due to the sample plus a relatively smooth background originating from the randomly structured water layer components (with some overall modulation due to the water-sample particle size). Such smooth backgrounds have been ameliorated or removed in SPI

previously[2–4] and a similar procedure may apply here, though note that the background here would also introduce coherent effects which may not necessarily be removed in an entirely equivalent manner. One drawback for this study, though perhaps of overall benefit, is that such a smooth background removal likely also ameliorates the contributions of inelastic scattering and radiation damage – at least to some extent. In practice, the effectiveness of such methods will depend on many experimental parameters, such as signal-to-background ratio in individual diffraction frames, number of data frames available, uniformity of the water later thickness across different instances and many more factors. This extended parameter space is the subject of a future work.

References

1. Hau-Riege, S. P., London, R. A., Chapman, H. N., Szoke, A. & Timneanu, N. Encapsulation and Diffraction-Pattern-Correction Methods to Reduce the Effect of Damage in X-Ray Diffraction Imaging of Single Biological Molecules. *Phys. Rev. Lett.* **98**, DOI: 10.1103/PhysRevLett.98.198302 (2007).

2. Rose, M. *et al.* Single-particle imaging without symmetry constraints at an X-ray free-electron laser. *IUCrJ* **5**, 727–736, DOI: 10.1107/S205225251801120X (2018).

3. Lundholm, I. V. *et al.* Considerations for three-dimensional image reconstruction from experimental data in coherent diffractive imaging. *IUCrJ* **5**, 531–541, DOI: 10.1107/S2052252518010047 (2018).

4. Quiney, H. M. & Nugent, K. A. Biomolecular imaging and electronic damage using X-ray free-electron lasers. *Nat. Phys.* **7**, 142–146, DOI: 10.1038/nphys1859 (2011).

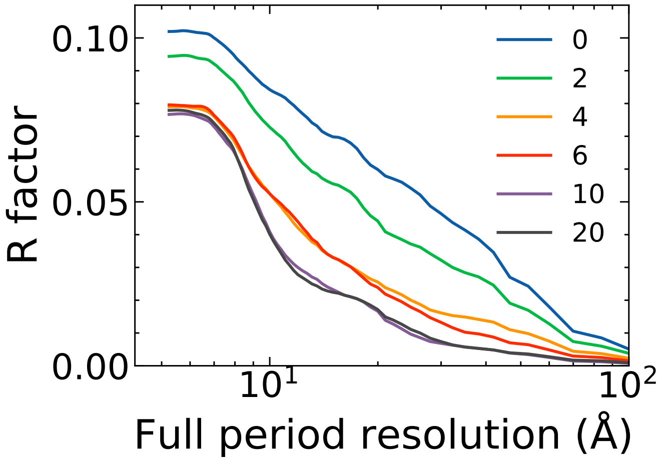

Figure S1. R factor calculated for diffraction patterns (including only elastic scattering from the protein) in case of the 'ionization damage'. During the calculation, the positions of all atoms were fixed to their initial values.

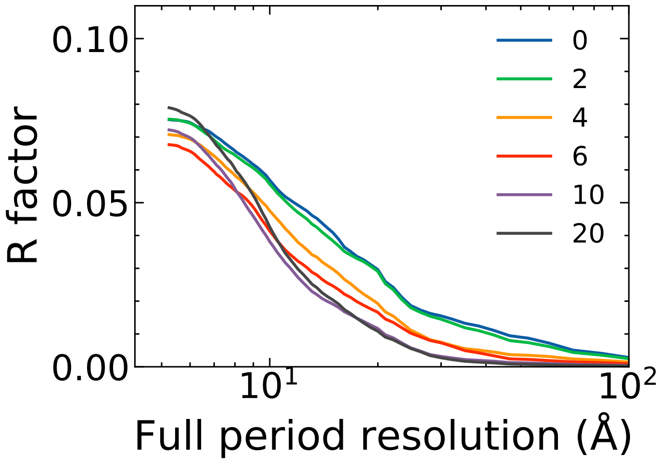

Figure S2. R factor calculated for diffraction patterns (including only elastic scattering from the protein) in case of the 'displacement damage'. During the calculation, atomic form factors were fixed to their initial values (corresponding to those of neutral atoms).

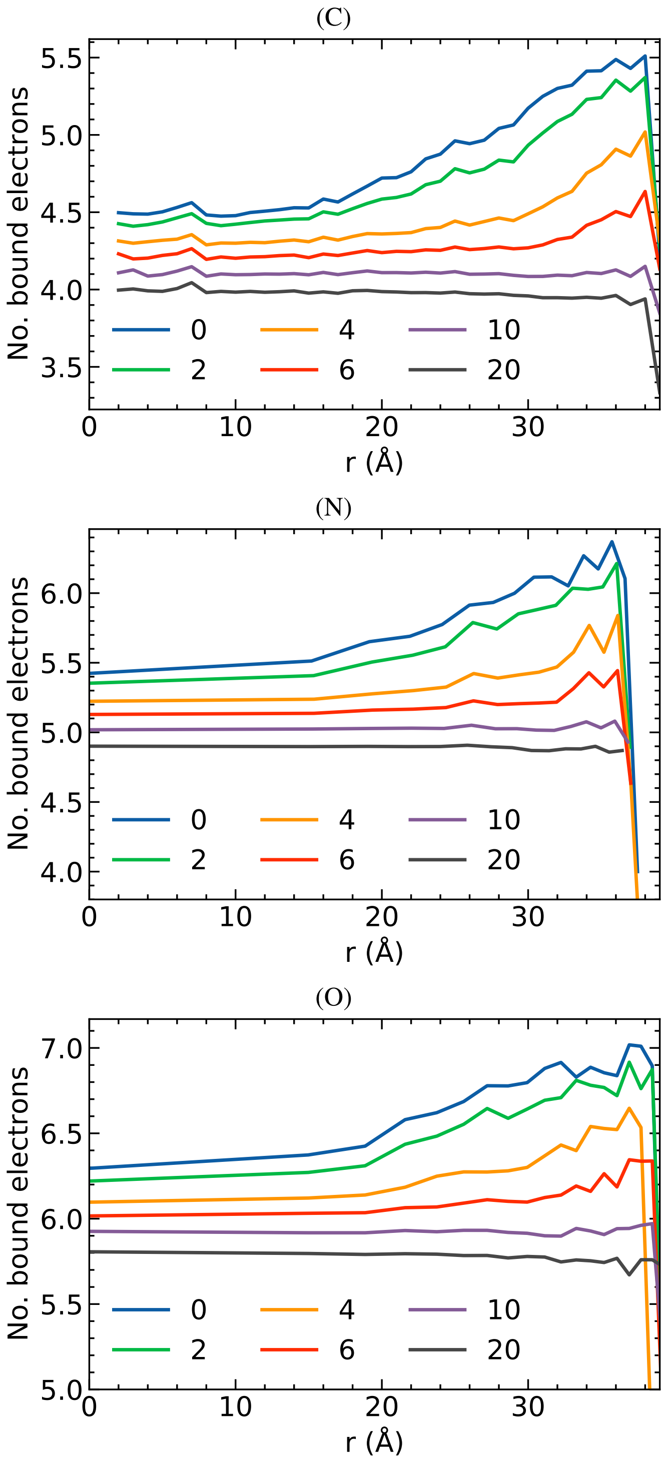

Figure S3. Radial profile of the average number of bound electrons for carbon, nitrogen, and oxygen atoms inside the irradiated 2NIP protein predicted at the time zero.

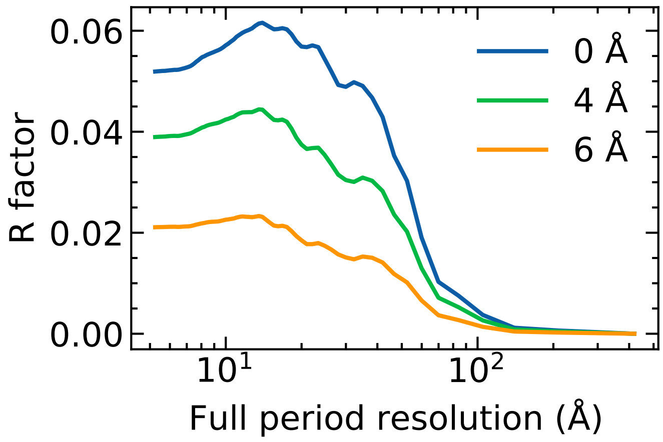

Figure S4. Effect of non-uniform distribution of the average number of bound electrons on R factor in low resolution region obtained for test samples with water thicknesses of 0 Å, 4 Å, and 6 Å.

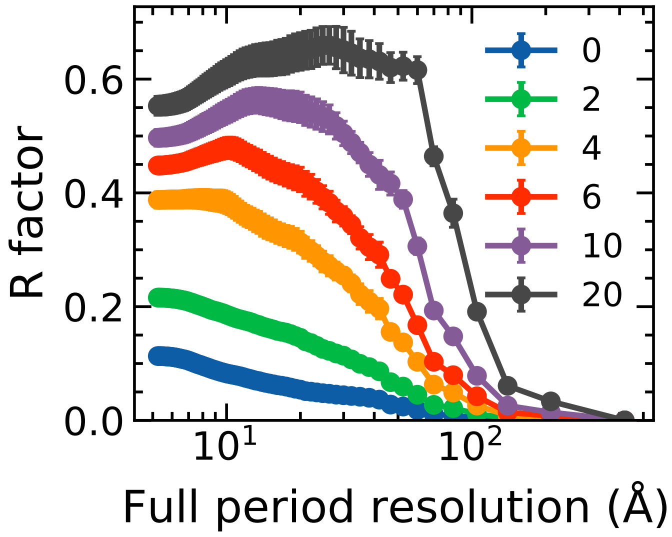

Figure S5. Measure of the diffraction pattern quality, R factor as a function of resolution. Both elastic scattering and inelastic scattering from both water and protein are considered. The error bars are the standard deviation from averaging over different orientations.
